# Mucosa-associated Lymphoid Tissue Lymphoma of Colon: A Case Report and Literature Review of Rare Entity

**DOI:** 10.7759/cureus.7438

**Published:** 2020-03-27

**Authors:** Navroop Nagra, Ajeet R Singhvi, Gaurav Singhvi

**Affiliations:** 1 Gastroenterology, Virginia Mason Medical Center, Seattle, USA; 2 Gastroenterology, Valley Endoscopy, Hemet, USA; 3 Gastroenterology, University of California, Los Angeles/David Geffen School of Medicine, Los Angeles, USA

**Keywords:** malt, rare cancers, colon cancer, gastrointestinal lymphomas

## Abstract

Mucosa-associated lymphoid tissue lymphoma (MALT lymphoma) accounts for approximately 5% of non-Hodgkin lymphomas, and the gastrointestinal (GI) tract is the most common site of involvement. The stomach and small intestine are the most common sites of involvement in the GI tract. Colonic MALT lymphoma is a rare condition that comprises only 2.5% of MALT lymphomas and less than 0.5% of all colon cancers. They usually present as colon mass or polyps. In this case report, we present a case of colonic MALT lymphoma diagnosed on random colon biopsies which is very rare.

## Introduction

Mucosa-associated lymphoid tissue (MALT) lymphoma, which is also referred to as extranodal marginal zone lymphoma, is a form of non-Hodgkin lymphoma (NHL) that predominantly involves the gastrointestinal tract [1,2]. The stomach is the most commonly affected site with MALT lymphoma [3]. The involvement of colon is very rare, and the presentation can vary from incidental finding on routine colonoscopy to severe gastrointestinal bleed [4]. Due to the rare occurrence of this condition, colonic MALT lymphomas are not well studied and there are no clear guidelines in terms of treatment. Herein, we report a case of incidentally diagnosed colonic MALT lymphoma in an asymptomatic patient during screening colonoscopy who ended up getting colectomy with no recurrence.

## Case presentation

**Figure 1:**
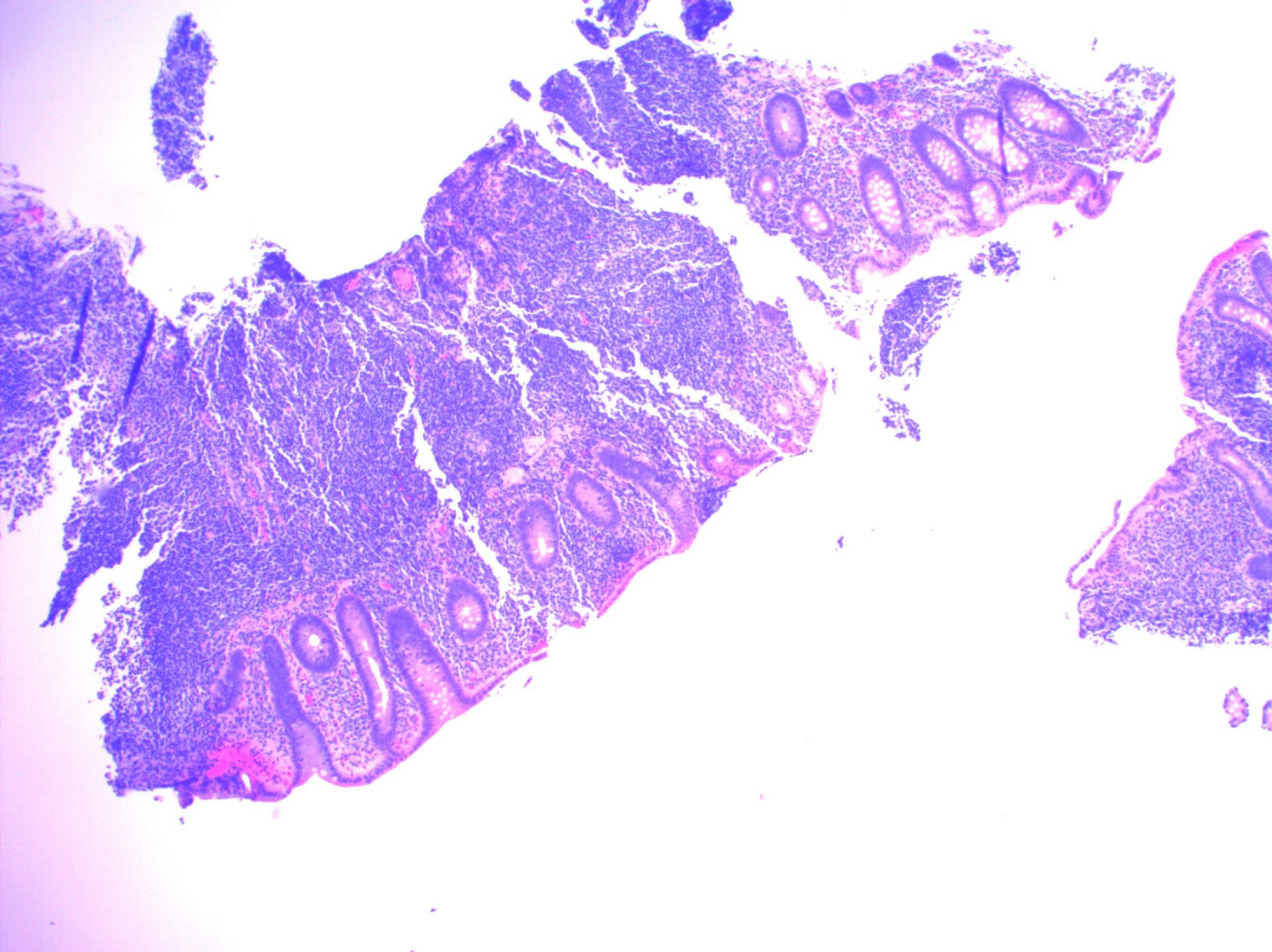
Dense and focally destructive lymphoplasmacytic infiltrate under low-power field (40x)

**Figure 2:**
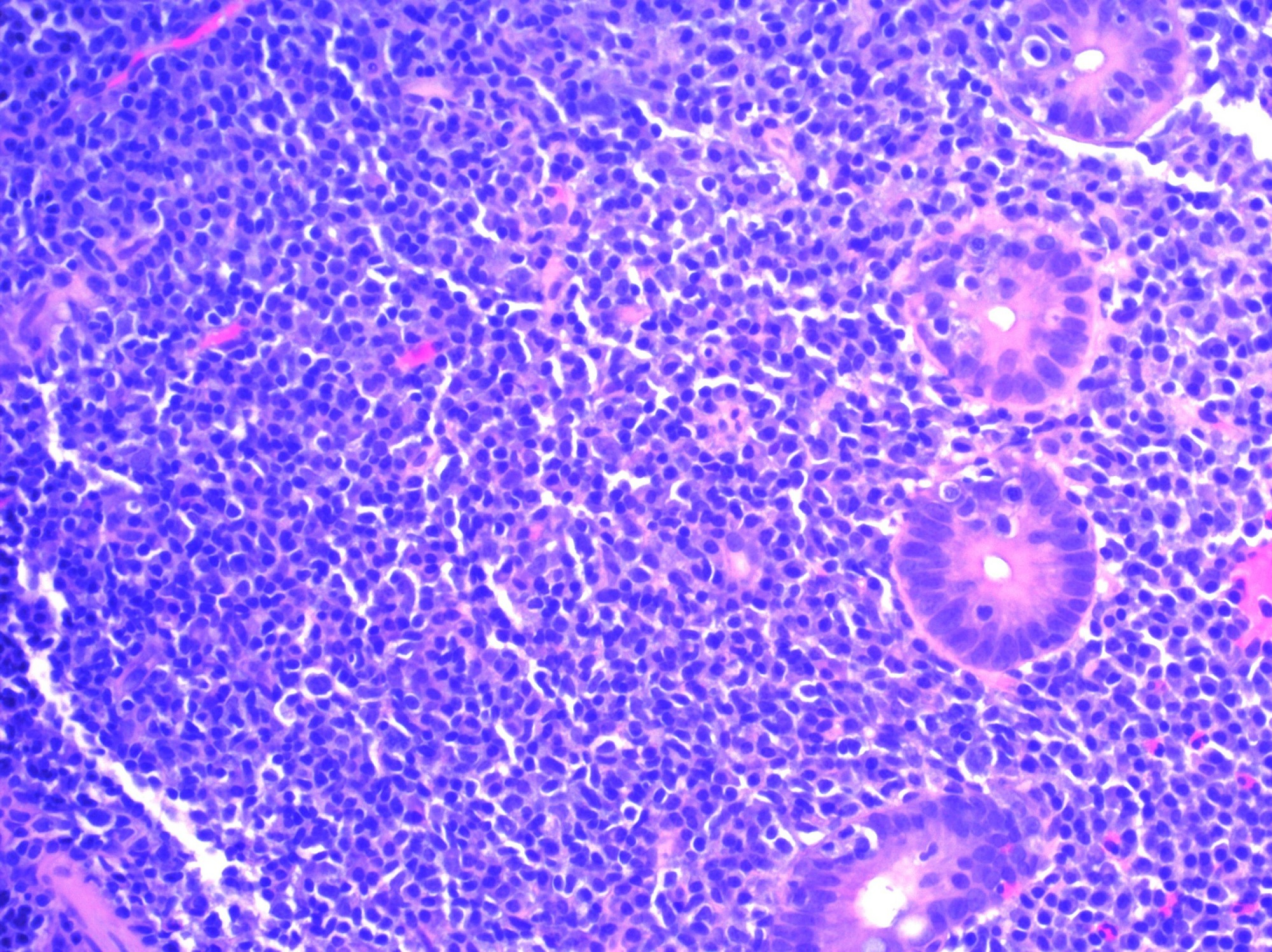
Dense and focally destructive lymphoplasmacytic infiltrate under high-power field (400x)

A 54-year-old female with a past medical history of hypertension underwent screening colonoscopy that revealed mild left-sided diverticulosis but was otherwise unremarkable. She was completely asymptomatic. No family history of colon cancer. Her recent blood work was unremarkable, and she had normal hemoglobin, normal white cell count, and platelets. Liver enzymes and renal function tests were also normal. During colonoscopy, random biopsies were taken from the cecum. To our surprise, pathology showed dense and focally destructive lymphoplasmacytic infiltrate which was positive for CD3, CD20, and CD43, consistent with MALT lymphoma (Figures 1, 2).

CT scan of the chest abdomen and pelvis did not demonstrate any suspicious lymph nodes or any signs of spread of lymphoma outside the colon. Repeat colonoscopy was performed after the biopsy results, random biopsies were taken from the rest of the colon, and there was no involvement of the left side. Esophagogastroduodenoscopy was also unremarkable. Biopsies taken on upper endoscopy and colonoscopy were negative for *Helicobacter pylori *(*H. pylori*). After a multidisciplinary team meeting with surgery and oncology, the final decision was to perform hemicolectomy without any need for adjuvant chemotherapy. She received a right hemicolectomy without any complications and recovered well. Subsequent imaging and colonoscopy one year later did not reveal any recurrence.

## Discussion

MALT lymphoma is a relatively uncommon subtype of NHL, which comprise 5%-10% of total NHL. It primarily involves the gastrointestinal tract but can also occur in lungs, ocular adnexa, and skin [5]. In the gastrointestinal tract, stomach is the most common site of involvement, followed by small intestine and colon, with gastric MALT lymphoma constituting about 70% of total cases. *Helicobacter pylor*i infection is strongly associated with gastric MALT lymphoma, but no association is seen between *H. pylori* and colonic MALT lymphoma. The mean age at diagnosis of MALT lymphoma is 60 years. Some studies show twice the incidence in females than males [6]. They arise from postgerminal center B cells, and most cases occur due to chronic immune stimulation by bacterial, viral, or autoimmune stimulus. Colonic MALT lymphoma only makes up 0.5% of colon cancers.

Most patients with colonic MALT lymphoma present with gastrointestinal bleed which can range from mild to massive [7]. It rarely can cause abdominal pain, perforation, intestinal obstruction, and intussusception. Some patients, like in this case, can be completely asymptomatic, and diagnosis is made on routine colonoscopy and biopsy of suspected site. Colonoscopic findings are nonspecific and can vary from single polypoid lesion to an ulcer or nodule to normal-appearing mucosa [8]. Immunophenotypic and genetic testing along with morphologic analysis of biopsy specimens is required for the diagnosis of MALT lymphoma. MALT lymphoma should be differentiated from other B-cell lymphoma like mantle cell lymphoma and follicular lymphoma and others. The typical morphological feature on biopsy is polymorphous infiltrate of small cells along with reactive-appearing follicles. Immunophenotypic features are most helpful in distinguishing MALT lymphomas from other lymphomas and malignancies. These cells express CD19, CD20, and CD20 B-cell markers [9]. The B cells in MALT lymphoma lack CD5, CD10, and bcl-2 rearrangements [10]. In terms of genetic analysis, detection of trisomy 3 or t (11; 18) is helpful in making the diagnosis. Once the diagnosis of MALT lymphoma is confirmed on histopathology, staging should be done. Although colonic MALT lymphoma can occur in isolation, there usually is simultaneous involvement of other areas of the gastrointestinal tract. Upper endoscopy, CT scan of chest, abdomen, pelvis, and bone marrow biopsy are needed for staging [11].

Due to the rare occurrence of colonic MALT lymphoma, there is no clear consensus on treatment. Surgical resection and chemotherapy have been widely utilized. There are rare case reports in which resolution occurred with *H. pylori* treatment [12,13]. Various chemotherapy agents used include chlorambucil, cyclophosphamide, vincristine, and prednisone. Rituximab as single-agent chemotherapy has been used in some cases with good response [14]. In this case, our patient underwent hemicolectomy and fortunately, there have been no signs of recurrence to date. She is under surveillance. 

## Conclusions

Our patient had a diagnosis of MALT lymphoma on random biopsies of the cecum and underwent right hemicolectomy without any chemotherapy. There are no signs of relapse to date. This case report emphasizes that colonic MALT lymphoma can present in various different ways, and since this condition is so rare, there are no standard guidelines for treatment. More data are needed to have definitive guidelines for the treatment of this rare disease.
